# There Are Two

**Published:** 1883-04

**Authors:** 


### "There are Two."

Dr. E. G. Betty, of this city, one of the rising young men of the profession, is not quite content to have all things go on in the common routine way. He has not exactly gone into fruit culture, but he has commenced raising pairs; or, to state it more definitely, two bright little girls came to his house a few days ago, and they insist on staying and making that their home, and, still more, they *will* call him Papa. And when they had been there fourteen days they were two weeks old.

CHANGE.—The following change in time of meeting will be of interest to the members of the profession in Georgia. By order of the President, and with the approval of the Executive Committee the next annual meeting of the Georgia State Dental Society has been changed from May, 1883, to August, 1883, in order to join with the Southern States Association. The joint meeting of these Societies will begin on the 2d Monday of August, 1883, at Atlanta, Georgia.

L. D. CARPENTER, Cor. Sec'y.

